# Dual role of brain-derived extracellular vesicles in dementia-related neurodegenerative disorders: cargo of disease spreading signals and diagnostic-therapeutic molecules

**DOI:** 10.1186/s40035-022-00326-w

**Published:** 2022-11-27

**Authors:** Francesca Natale, Salvatore Fusco, Claudio Grassi

**Affiliations:** 1 Department of Neuroscience, Università Cattolica del Sacro Cuore, 00168 Rome, Italy; 2 Fondazione Policlinico Universitario A. Gemelli IRCCS, 00168 Rome, Italy

**Keywords:** Extracellular vesicles, Neurodegenerative diseases, Stem cells, Biomarkers, Cognitive decline

## Abstract

Neurodegenerative disorders are one of the most common causes of disability and represent 6.3% of the global burden of disease. Among them, Alzheimer’s, Parkinson’s, and Huntington’s diseases cause cognitive decline, representing the most disabling symptom on both personal and social levels. The molecular mechanisms underlying the onset and progression of dementia are still poorly understood, and include secretory factors potentially affecting differentiated neurons, glial cells and neural stem cell niche. In the last decade, much attention has been devoted to exosomes as novel carriers of information exchanged among both neighbouring and distant cells. These vesicles can be generated and internalized by different brain cells including neurons, neural stem cells, astrocytes, and microglia, thereby affecting neural plasticity and cognitive functions in physiological and pathological conditions. Here, we review data on the roles of exosomes as carriers of bioactive molecules potentially involved in the pathogenesis of neurodegenerative disorders and detectable in biological fluids as biomarkers of dementia. We also discuss the experimental evidence of the therapeutic potential of stem cell-derived vesicles in experimental models of neurodegeneration-dependent cognitive decline.

## Background

### Neurodegeneration and dementia

Neurodegenerative diseases like Alzheimer’s (AD), Parkinson’s (PD), and Huntington’s (HD) are a heterogeneous group of neurological disorders characterized by progressive derangement of neuronal structures and functions in both central and peripheral nervous systems [1, 2]. A combination of genetic factors and environmental conditions plays a crucial role in their pathogenesis, and the numerous underlying molecular mechanisms, far from being fully elucidated, make them some of the most difficult diseases to diagnose and treat [3]. More importantly, to date, no definitive cure has been discovered for most of these pathologies and the current therapeutic approaches available only aid in relieving symptoms. At cellular level, they are characterized by neuronal dysfunction eventually leading to cell death, markedly affecting the ability of different brain areas (e.g., neocortical and hippocampal regions in AD and striatal regions in PD and HD) to communicate [4]. Common pathogenic mechanisms theorized to be involved in these neurodegenerative disorders include neuroinflammation, dysregulation of intracellular protein trafficking and endocytic sorting, oxidative stress, aggregation and degradation of defective proteins and formation of inclusion bodies, mitochondrial dysfunction, generation of reactive oxygen species (ROS), iron accumulation, and epigenetic alterations [5–8]. The exact sequence of molecular events leading to the development and progression of neurodegenerative diseases is far from being fully understood. Moreover, the pathogenic role of protein aggregation in human sporadic neurodegenerative diseases remains controversial [9]. Different items of evidence suggest that misfolded molecules and protein aggregation can either contribute to the propagation of neurodegeneration and be convergent drivers of cellular death or represent epiphenomena of multiple diseases, or even act as compensatory responses to cellular stress in the attempt to sequester misfolded proteins as a protective mechanism [10–12]. However, it is conceivable that, in some brain areas, a sort of vicious circle among several components that fuel the progression of neurodegeneration is established. Age-related epigenetic changes and loss of proteostasis can lead to autophagy alteration and mitochondrial dysfunction, causing the increase of oxidative stress and abnormal protein aggregation [13]. Oxidative stress also contributes to protein misfolding and aggregation by altering proteosome pathways [14] and, in turn, accumulation of protein oligomers and aggregates may impair mitophagy and enhance the formation of oxidative species and neuronal death [15, 16]. In addition, neuroinflammation and aberrant communication among microglia, other glial cells and neurons may promote protein misfolding and neuronal apoptosis and play a critical role in the progression of neurodegeneration [17–19]. These molecular alterations lead to a progressive deterioration of intellectual functions and, eventually, to the development of dementia-related symptoms such as memory deficits and decline in understanding and reasoning.

Thus, aberrant cell-cell communication occurring inside the brain can contribute to the onset and progression of neurodegenerative disorders. Extracellular vesicles (EVs) have been identified as carriers of molecules participating in the signal exchange among brain cells [20]. Isolation of circulating EVs from biological fluids is emerging as a novel liquid biopsy modality providing a plethora of biomarkers for human pathologies such as cancer and cardiovascular diseases [21]. More recently, they have attracted the interest of neuroscientists for their potential role in the spreading and diagnosis of neurodegenerative disorders [22]. Indeed, the lipid bilayer membrane structure of these vesicles makes their cargo more stable and potentially available for intercellular communication inside the brain, and offers a source of minimally invasive biomarker detection for early diagnosis and real-time monitoring of neurodegeneration progression. Moreover, recent data suggest that stem cell-derived EVs may represent novel therapeutic tools against neurodegenerative disorders because of their biocompatibility, and ability to cross the blood-brain barrier and target brain tissues [23]. From the therapeutic perspective, EVs derived from stem cells represent a promising tool as their cargo contains molecules potentially fostering the pro-regenerative and immunomodulatory pathways activated in their parental cells [24]. Indeed, molecules released from stem cells through EVs can exert therapeutic effects in regenerative medicine and tissue repair as well as in the modulation of immune responses [25].

Thus, the rising attention to EVs in the field of neurodegeneration is due to multiplex aspects including their potential role in the pathogenesis, diagnosis, and therapy.

Here, we review the role of EVs as carriers of molecules potentially involved in the protein aggregate spreading/pathogenesis of some major dementia-related neurodegenerative disorders, i.e., AD, PD and HD. Furthermore, we summarize the most recent discoveries about the identification of EV-derived molecules as potential biomarkers for diagnosis and prognosis of these diseases. Finally, we describe evidence on the therapeutic potential of stem cell-derived EVs to counteract cognitive decline and restore brain functions in experimental models of neurodegenerative diseases.

### AD

AD is a neurodegenerative, progressive and irreversible brain disease that affects cognitive function, personality and behavior and it is the most common cause of dementia in the elderly population [26]. AD is characterized by abnormal synaptic functions due to a plethora of pathogenic mechanisms including mitochondrial dysfunction, neuroinflammation and oxidative stress that cause alterations of protein homeostasis and lead to development of AD molecular hallmarks such as deposition of both amyloid-β (Aβ) and phosphorylation of microtubule-associated protein Tau (i.e., neurofibrillary tangles, NFTs) in the brain [27, 28]. Intra- and extra-cellular protein aggregates lead to a reduction in the number and function of synapses and, consequently, to deficits of synaptic transmission and plasticity [29, 30]. The increased Aβ peptides aggregate into soluble oligomers and activate microglia to enhance inflammatory responses and mitochondrial damage. At the same time, the abnormally phosphorylated Tau protein can form NFTs, which leads to neuronal apoptosis and induces a decrease in synaptic function [31]. In addition, dysfunction of both autophagy mechanisms and endosomal-lysosomal system, which has been shown to play a role in the formation of the amyloid plaques, has been linked to AD pathogenesis [32].

### PD

PD is the second most common neurodegenerative disease in the world after AD [33]. The main pathological hallmarks consist in the degeneration and death of dopaminergic neurons, presence of eosinophilic inclusion bodies (i.e., Lewy bodies) in the cytoplasm of residual neurons in substantia nigra and a significant decrease of dopamine in the striatum. Evidence from genetic and biochemical studies supports a key role of the nerve terminal protein α-synuclein in the pathogenesis of PD. Mutations and multiplications of the α-synuclein gene (*SNCA*) are known as a cause of familial PD, and genome-wide association studies have identified mutations and polymorphisms in the *SNCA* locus (e.g., *SNCA* rs356219 A/G polymorphism) as potential risk factors [34, 35]. More recently, the spread of pathological forms of α-synuclein from one cell to another is increasingly considered a key player in the progression of synucleinopathies [36]. Toxic forms of α-synuclein spread and deposit among a variety of cells, such as astrocytes and microglial cells, triggering inflammation and leading to degeneration of neurons and exacerbation of PD [37–39].

### HD

HD is an autosomal dominant neurodegenerative disorder caused by an expansion of CAG repeat in the huntingtin gene on chromosome 4 [40–42]. This mutation produces an extended N-terminal polyglutamin stretch in the huntingtin protein (HTT), leading to intracellular accumulation and aggregation of this protein. Accumulation of mutant huntingtin (mHTT) aggregates causes mitochondrial dysfunction, striatal cell death through transcriptional dysregulation, activation of apoptosis pathways, and alterations of physiological protein-protein interaction [43]. While mHTT spreading is sufficient to induce pathological changes, it is not yet clear whether protein spreading significantly contributes to HD onset and progression given the ubiquitous expression of mHTT. Anyway, several studies have reported how the spreading of mHTT between cells triggers HD-related behaviors and pathologies [44].

### EVs

A plethora of EVs circulate in different biological fluids including blood and cerebrospinal fluid (CSF). They differ in size, density, and composition. These vesicles, surrounded by a lipid bilayer membrane, are classified depending on their originating pathway. They are released by multiple cell types and carry a variety of cargo molecules including coding and noncoding RNAs, lipids and proteins. Based on their size/shape, as well as site and mechanisms of biogenesis, EVs are classified as (1) ectosomes, which include microvesicles and oncosomes (50–10,000 nm) that are generated by the outward budding of plasma membrane, (2) migrasomes (500–3000 nm), which are released during cell migration, (3) exopheres (1000–10,000 nm) and amphisomes that are likely related to macroautophagy, (4) apoptotic bodies (50–5000 nm), which are released from apoptotic cells, (5) exosomes (30-150 nm) that are generated from endosomal membrane as intraluminal vesicles, and (6) exomeres (<50 nm) and retroviral-like particles that are of uncertain origin. Some types of vesicles partially overlap in terms of cargo composition and size, while differing in the site of biogenesis. Of note, depending on the type of cell where the EVs come from, the biogenesis, the mechanisms of secretion and the cargo composition of the same subgroup of vesicles may vary [45]. EVs, which can travel long distances to deliver their content to target cells, are able to both modulate physiological cellular activities in host cells and carry a trace of donor cells potentially resembling the molecular changes occurring in the tissue where they are generated. Although they were originally considered as cell debris, in the last two decades EVs are emerging as a source of pivotal signals for intercellular communication [25, 46]. In addition to classic routes of communication (e.g., secretion of autocrine and paracrine signaling factors), EVs represent an additional strategy for cells to release signals in the surrounding cellular environment or to deliver molecules to distant tissues [25, 47]. In the central nervous system (CNS), cell-to-cell interaction is essential for development, brain plasticity, and metabolic homeostasis, and its alteration is critically involved in the development of neurodegenerative diseases [48]. The molecular cargo of EVs contains various bioactive molecules originating from different cell compartments (i.e., cytoplasm, plasma membrane, mitochondria, and nucleus), including enzymes, receptors, growth factors, transcription factors, lipids and nucleic acids (mRNAs, miRNAs, and DNA). On the other side, EV membrane is composed of different lipids and surface proteins, such as tetraspanins, fusion and transferring proteins, lysosome-associated membrane glycoproteins, heat shock proteins, cytoskeleton proteins, integrins, transferrin receptors and MHC molecules [49–52].

Among the different types of EVs, exosomes are certainly the most studied. Exosomes originate from the multivesicular bodies (MVBs) and are released in the extracellular space upon fusion of endosomes with the plasma membrane of the donor cell. Exosomes are involved in physiological dynamics such as homeostasis maintenance, neurogenesis, synaptic plasticity, myelination, neuron survival and regeneration upon brain injury [53–56]. Neurons release exosomes containing neurotransmitter receptors and activity-related miRNAs that can influence the excitability and synaptic plasticity of neighboring cells [57, 58]. Neuronal EVs are also able to transfer into astrocytes the miR124a, which modulates glutamate uptake by regulating the expression of excitatory amino acid transporter 2 [59]. Glial cell-derived EVs are involved in the modulation of neuronal activity, repair and response to cellular stress. For instance, astrocyte-enriched cultures release synapsin via exosomes, which promotes neurite outgrowth [60]. In addition, the glutamate-dependent crosstalk between neurons and oligodendrocytes induces the release of oligodendroglial exosomes containing neuroprotective RNAs and proteins under oxidative stress conditions [61, 62]. Moreover, Schwann cell-derived exosomes have been demonstrated to facilitate axonal elongation in vitro and sustain nerve fiber regeneration in vivo [63]. To date, very little is known about the mechanisms regulating the targeting of EVs and if vesicles generated from some donor cells are preferentially transferred to specific target cell types [64]. Rana et al. have highlighted the critical role of exosomal tetraspanin-complexes to influence target cell selection through interactions with the integrin protein family both in vivo and in vitro [65].

In addition to their role in the intercellular crosstalk among brain cells, EVs may also allow the removal of unwanted proteins and other macromolecules from the cell [66]. Indeed, EVs can eliminate toxic aggregated proteins when the autophagic–lysosomal pathway becomes insufficient. For instance, exosomal secretion acts as a compensatory pathway for the clearance of cellular TDP-43 aggregates in Neuro2a cells [67]. In addition, other disease-causing toxic misfolded proteins are exported from neurons through vesicle secretion, reinforcing the idea of an off-site disposal strategy [68]. For instance, Braun’s group demonstrated that both mutant superoxide dismutase-1 and polyglutamine-expanded HTT are exported via EVs from catecholaminergic derived CNS cells, with the help of the molecular chaperone CSPα [69]. Moreover, neuronal exosomes have been reported to facilitate conformational change of extracellular Aβ into nontoxic amyloid fibrils and promote its internalization by microglia for degradation [70]. Furthermore, the proteolytically active insulin degrading enzyme, which targets and degrades also Aβ peptides, is encapsulated in exosomes and released in the extracellular space, thereby promoting Aβ catabolism [71]. Thus, EVs can influence brain health and plasticity via the exchange of signals regulating neuronal activity, metabolism, and axon regeneration, but their cargo can also target different pathways potentially involved in the onset and progression of neurodegenerative disorders.

## Role of brain-derived EVs in the spreading of neurodegeneration

**Fig. 1:**
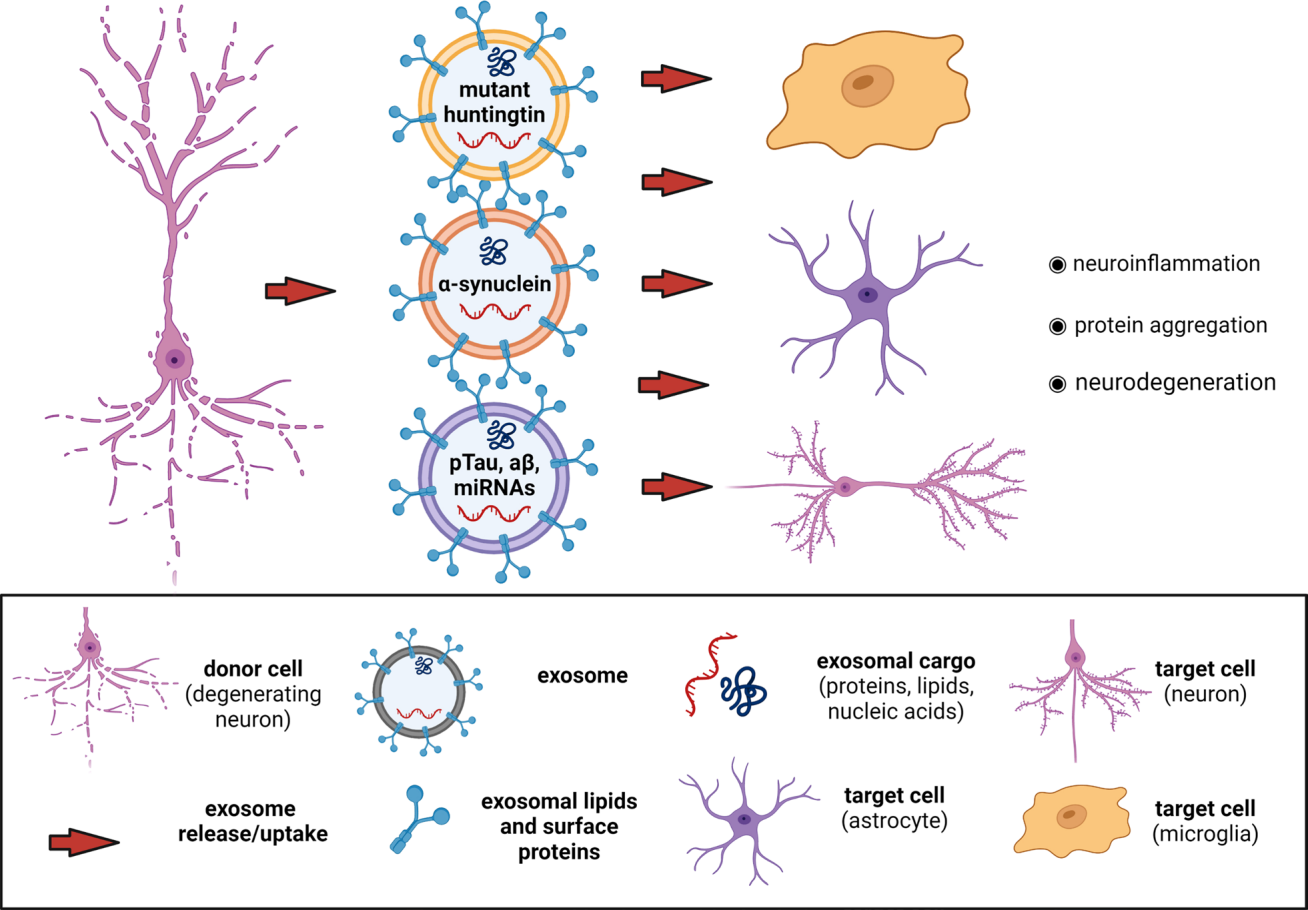
Role of brain-derived exosomes in the spreading of pathology in neurodegenerative diseases. HD, Huntington’s disease; PD, Parkinson’s disease; AD, Alzheimer’s disease

Apart from their physiological contributions to cell-cell communication, EVs have recently emerged as a carrier of molecules, contributing to the spreading of neurodegenerative disorders in the CNS (Fig. 1). Brain-derived exosomes (BDEs) appear to potentially contribute to the onset and progression of neurodegeneration mainly through the transport of synaptotoxic proteins triggering glial cell activation and neuroinflammation [72]. In addition, exosomes released from activated glial cells can incorporate pro-inflammatory molecules, such as cytokines [39], or abnormally expressed miRNAs that, once transferred to target cells, can trigger epigenetic dysregulation of brain plasticity-related gene expression, and promote the development of neuroinflammation and neurodegeneration [73]. Along with these mechanisms, EVs can promote extracellular spreading of pathological misfolded and aggregated proteins [74–76]. Of course, the neurotoxic misfolded proteins involved in neurodegenerative diseases such as α-synuclein, Aβ and Tau, can also extracellularly diffuse and spread from affected cells to naive cells where they template aggregation through other mechanisms including in a prion-like manner [77, 78]. For example, lysine residues mediate the interaction between Tau and the transport protein LRP1, mediating Tau endocytosis and its spread [79]. In addition, Tau can be intercellularly transferred through tunneling nanotubes, which are filamentous actin-containing channels that connect neighboring cells [80]. Finally, it has been demonstrated that hyperphosphorylated tau can be directly secreted as a naked protein from the cells [81, 82]. Moreover, a recent study reported how microglia may phagocytose Aβ and contribute to the propagation of Aβ pathology by invading non-diseased brain tissues [83]. Below we summarize the evidence regarding the role of BDEs in the propagation of pathological molecules triggering the progression of AD, PD, and HD.

### AD

#### Tau pathology

Tau is a microtubule-associated protein, predominantly expressed in neurons. It is released from neuronal cells by several mechanisms: (1) an activity-dependent release [29], (2) bursting of apoptotic neurons, resulting in release of free Tau molecules in the extracellular space, which are then internalized by neighboring cells, contributing to the propagation of Tau pathology, (3) sorted and packed Tau proteins in EVs released by exocytosis [84]. Encapsulation of Tau by murine exosome-like EV membranes has been demonstrated to induce Tau aggregation [85]. Since Tau aggregation is thought to be driven by post-translational modifications, it is relevant to understand how exosomal and cytoplasmic Tau differ in terms of phosphorylation patterns [76, 86]. Interestingly, exosomes isolated from the CSF of patients with mild (Braak stage 3) and established neurodegeneration (Braak stage 5) are strongly positive for Tau protein, specifically Tau phosphorylated at threonine 181, and the proportion of pTau in the exosomal fraction is significantly higher in mild/moderate AD groups than in controls [76]. This evidence suggests that Tau modified with particular phospho-signatures may preferentially be targeted into exosomes [87, 88]. Moreover, the tyrosine kinase protein Fyn, which can further contribute to the phosphorylation of the vesicle-encapsulated Tau proteins, is detected inside the exosomal cargo [76].

The pathogenesis of tauopathies involves conversion of Tau monomers into pathological Tau conformers that serve as templates to recruit native Tau into growing assemblies. Small soluble Tau seeds have been proposed to drive pathological Tau assembly in vitro and in vivo [89, 90]. In addition to differences in phosphorylation sites, the seeding competency also varies among different tauopathies [91]. In recent years, many efforts have been made to study tau pathobiology [92, 93]. The internalization of exosomal Tau seeds appears to be quite complex, given that cells internalize EVs by a variety of endocytic pathways, including clathrin-dependent and -independent pathways such as caveolin-mediated uptake, macropinocytosis, phagocytosis, and lipid raft-mediated internalization [94]. From exosomes, Tau oligomers are released to the cytosol of the recipient neuron, where they form NFTs with other Tau oligomers [95, 96]. In addition, different cell types contribute to the progression of tauopathy. Apart from neurons, microglia can phagocytize Tau-containing neurons and secrete exosomes containing Tau, which facilitate its propagation into neurons. Accordingly, both depletion of microglia and inhibition of exosome synthesis counteracte Tau propagation in a mouse model of AD [97].

#### Aβ pathology

Aβ peptides are produced from amyloidogenic cleavage of amyloid protein precursor (APP) and can aggregate to form soluble toxic species that cause synaptic dysfunction and memory deficits in brains of AD patients [98–100]. Although exosomes represent an essential route for clearance of Aβ from the cell, they might also represent a source of Aβ aggregates for the nearby cells, contributing to Aβ aggregation [70]. The main site of Aβ production in neurons is the MVBs [101]. MVBs may be degraded upon delivery to the lysosomes or secreted as exosomes through fusion with the plasma membrane [102], consequently spreading their content. The extraction of Aβ oligomer-rich exosomes from AD brains demonstrates that exosomes could carry Aβ aggregates among neurons [103], triggering neuroinflammation and accelerating amyloid plaque formation within the brain [104]. Accordingly, exosomal proteins such as Alix and flotillin-1 have been found to be enriched around amyloid plaques in the brains of AD patients [74]. Moreover, presenilin, APP, C-terminal fragments of APP (APP-CTFs), and several key proteases involved in Aβ production such as β-secretase (BACE1) and γ-secretase (presenilin subunits PS1 and PS2) have also been found in exosomes isolated from AD brain cells/tissues [105–107].

Finally, microglia hyper-secrete exosomes that contribute to Aβ oligomerization and deposition as well as tau propagation, while depleting microglia halts this effect [108]. Blocking exosome secretion/formation or their uptake by brain cells might reduce the spread of Aβ oligomers and thus alleviate Aβ toxicity. Accordingly, reduction of secreted exosomes induces a decrease in amyloid plaque deposition in the 5×FAD mouse model [109].

#### Other mechanisms

Besides by contributing to Tau and Aβ propagation, microglia- and astrocyte-secreted exosomes participate in the progression of AD also by release of neuroinflammatory molecules and non-coding RNA species. For instance, exosomes released from APP-overexpressing cells are internalized by microglia and induce microglial activation and release of pro-inflammatory cytokines [110, 111]. Moreover, the interaction between released miRNAs and toll-like receptors (TLRs) can trigger neuroinflammation and neurodegeneration. The secretion of miRNA let-7 into the extracellular environment has been demonstrated to stimulate the RNA-sensing protein TLR7 in both microglial and neuronal cells, and its intrathecal injection results in neurodegeneration in wild-type mice [112]. Finally, exosomes released by activated microglia are reported to spread the inflammatory milieu composed of proteins involved in cell metabolism, autophagy-lysosomal pathway, and cell matrix reorganization, which modulate astrocyte activity in vitro [113].

### PD

Recently, several in vivo studies have provided evidence of exosome involvement in the progression of PD pathology. Exosomes derived from CSF of patients with PD contain pathological forms of α-synuclein, which induce oligomerization of soluble α-synuclein in recipient cells in a dose-dependent manner [114]. Accordingly, exosomal α-synuclein can spread more efficiently from a donor cell to target cells compared to exosome-free α-synuclein in naïve human neuroglioma H4 cells in vitro [115]. In addition, exosomes accelerate the aggregation of exogenous α-synuclein and conversion from monomeric α-synuclein into fibrillar aggregates [116].

Microglia, the innate immune cells inside the brain, endocytose aggregated forms of α-synuclein released from neurons, in an attempt to clear pathogenic species of the protein [117]. However, excessive α-synuclein uptake in glial cells can produce protein aggregates similar to those found in PD brains and trigger inflammatory responses, leading to the incorporation of fibrils into exosomes [118, 119]. The interaction of exosomal α-synuclein and microglial TLR2 has emerged as an important driving force for excessive microglial phagocytosis of α-synuclein, and exosomes derived from microglia have been suggested to be responsible for cell-to-cell transmission of pathological forms of α-synuclein, promoting the spread of PD [120]. Indeed, intrastriatal injection of microglia-derived EVs can transfer exosomal α-synuclein to neurons in vivo [121]. Accordingly, stereotaxic injection of microglia-derived exosomes containing α-synuclein promotes the spreading of aggregates throughout several brain regions. Depletion of resident microglia decreases the transmission of α-synuclein, indicating the critical contribution of microglia and microglia-derived exosomes to the pathology [120]. Recent in vitro studies demonstrated that the treatment of primary cultures of mouse microglia with α-synuclein preformed fibrils induces the release of exosomes containing pathogenic α-synuclein forms, which trigger α-synuclein aggregation in recipient neurons. In addition, it has been reported that α-synuclein released from injured neurons is able to induce and maintain inflammatory responses through activation of glial cells [38]. Indeed, like a positive feedback loop, the activated glia drive protein aggregation, which in turn propagates neuroinflammation.

### HD

Small EVs are involved in mHTT propagation between cells at both protein and RNA levels. mHTT protein carrying expanded polyglutamine sequence is prone to abnormal conformation, and both post-translational modifications and protease cleavage may contribute to the formation of a misfolded N-terminal fragment. This protein tail, when unsuccessfully re-folded, may accumulate, engulfing the ubiquitin-proteasome and autophagy system and, eventually, be released by exosomal incorporation and secretion [122]. Despite being a large 360-kDa protein, the full-length HTT was co-isolated with EVs from HD patient plasma in a recent study, suggesting the presence of full-length, fragmented and aggregated forms of both mutant and wild-type HTT in small EVs [123]. In addition, human HEK293T cells infected with a lentivirus encoding mutant *HTT-*exon 1 fragments generate EVs containing toxic expanded trinucleotide repeat RNAs (CAG-repeat RNA) and polyQ protein [44, 124]. Similarly, murine embryonic fibroblasts overexpressing the exon 1 of the *Htt* gene showed constitutive interaction of mHTT with exosome structural proteins Alix and TSG101 [125]. In both primary cultured astrocytes and the striatum tissue from an HD mouse model, mHTT impairs exosome secretion by decreasing the expression of the glial protein αB-crystallin, a heat shock protein mediating exosome release, leading to defective exosome secretion and accumulation of mHTT aggregates within the cell [126]. Several studies suggested that exosomes can transport the expanded polyglutamine tract of mHTT RNA and protein, as well as mHTT aggregates. The transport of mHTT into exosomes and the ability of the latter to transfer mHTT between cells have been confirmed by detection of mHTT aggregates in neurons derived from wild-type neural stem cells (NSC) co-cultured with HD fibroblasts. Strikingly, the injection of exosomes released from fibroblasts of HD patients into a newborn mouse brain triggers the manifestation of HD-related behavior and pathology, characterized by motor and cognitive deficits [44].

These data highlight the overall contribution of exosomes to the pathogenesis and progression of different neurodegenerative disorders, targeting multiple cells involved in both synthesis and clearance of misfolded neuropathological protein aggregates.

## Brain-derived exosome cargo as a source of biomarkers

### Brain-derived exosomes

Disease biomarkers are important for early diagnosis and follow-up of human pathologies and they represent a valuable tool for personalized medicine. Although currently there are no highly validated nanovesicle-derived biomarkers for diagnosis and monitoring of neurodegenerative diseases, more attention is being paid to exosomes and their content as a promising source of molecules with great potential to study brain disorders, mainly because of the current lack of other specific and non-invasive biomarkers [127–129]. As previously described, exosomes are released from numerous cell types and found in biological fluids, including blood, urine, saliva, breast milk, CSF, semen, amniotic fluid, and ascites, making them attractive for use through liquid biopsies. Even though the isolation techniques still need to be refined to reach an adequate level of both specificity and reproducibility for clinical setting, currently vesicles can be isolated from biological fluids to analyze their cargo as a molecular trace of the cell from which they are generated, therefore providing a fingerprint of physiological and pathophysiological status of parental cells [130]. Exosomes are already being considered as a source of biomarkers for the diagnosis of cancer as well as for cardiovascular diseases [131–133]. BDEs can be isolated from the CSF [134] but they can also cross the blood-brain barrier (BBB) and be detected in peripheral body fluids, thus overcoming the limits of accessibility to the CNS [135]. In recent years, numerous studies have reported the possibility to enrich neuronal or glial BDEs starting from biological fluid samples (e.g., blood) [136–138]. Numerous methods for exosome isolation have been set up in the most recent years, ranging from filtration and ultracentrifugation to microfluidics array [139, 140]. Unfortunately, only few of them allow extraction of enough numbers of exosomes starting from patient samples. In 2015 two groups succeeded in isolating CNS-derived exosomes (i.e., neuronal and astrocytic vesicles) from blood plasma through polymer-assisted precipitation followed by immunoprecipitation with L1CAM (or superparamagnetic L1CAM-conjugated microbeads immuno-capture) or GLAST antibodies, respectively [141–143]. A few years later, Dutta and colleagues were the first to successfully isolate oligodendrocyte-derived exosomes from blood by using a MOG antibody coupled with magnetic dynabeads [144]. The main challenge remains to confirm if the isolated exosomes indeed originate from the intended cell type. For example, it is important to consider that L1CAM is not exclusively expressed in neurons. On the other hand, proteomic analyses showed that L1CAM-captured exosomes contain higher concentrations of CNS-derived proteins (e.g., pTau, microtubule associated protein 2, neurofilament light chain, and L1CAM) than total exosome samples [145]. However, unanimous consensus has not yet been reached on the specificity of BDE isolation using these experimental approaches. At present, nearly 30 registered clinical trials on “clinicaltrials.gov” are investigating the potential of EVs as a source of biomarkers related to specific diseases, including obesity, cancer, cardiovascular diseases, and neurodegenerative disorders.

### AD

Specific changes in exosomal cargo and transmembrane proteins are reported to be significantly related to AD onset and progression, and they can provide useful and valuable biomarkers. Exosomes can carry Aβ1–42, Tau and its phosphorylated forms (e.g., p-Tau^thr181^ and p-Tau^ser396^), lncRNAs, miRNAs and other proteins that constitute molecular hallmarks of AD and could help distinguish AD or mild cognitive impairment (MCI) patients from healthy individuals [146, 147]. As the pathology progresses from preclinical stages through MCI to dementia, the concentrations of these molecules in biological samples change, along with increased expression of other risk factor-associated molecules which are involved in neuroinflammation (C1q), autophagy system dysregulation (cathepsin-D) and metabolic disorders (IRS-1 and p-IRS-1) [148]. Of all, neuron-derived exosomes (NDEs) present the highest diagnostic relevance compared to total exosomes, especially when considering p-Tau levels [129]. In addition, combination of multiple markers (CSF p-Tau and Aβ) may increase the sensitivity and specificity. For instance, Aβ1–42 levels in neuron-derived blood exosomes in patients with preclinical AD are significantly higher than those from healthy control subjects, but significantly lower than those from AD patients [141]. Along with protein analysis, characterization of exosomal miRNA profile can provide accurate insights into the pathogenesis and progression of the disease. Alterations of exosomal miRNAs from other body fluids, including plasma and CSF, have been reported by several studies. For example, analysis of CSF-derived exosome samples reveals alterations of miR-16-5p, miR-125b-5p, miR-451a, miR-605-5p, miR-9-5p and miR-598 in AD patients when compared to healthy subjects [149]. Many of these miRNAs have been shown to be implicated in AD pathogenesis [150]. Moreover, analysis with deep sequencing techniques of exosome-enriched plasma fractions reveals increased expression of 20 plasma exosomal miRNAs in AD patients. Among these 20 miRNAs, a panel of 7 (miR-185-5p, miR-342-3p, miR-141-3p, miR-342-5p, miR23b-3p, miR-338-3p, and miR-3613-3p) is highly valuable for predicting AD status with high accuracy using a machine learning model [151]. Finally, more exosome-based biomarkers for both preclinical and clinical AD are emerging. Indeed, the blood exosomal levels of BACE1-antisense transcript (BACE1-AS), NDE APP, APPα, and APPβ are reported to be significantly higher in AD patients compared to control subjects [143, 152]. Of note, free plasma proteins such as phospho-Tau217 and GFAP have recently been reported to be useful biomarkers of early AD, even before neuroimaging alterations can be detected [153, 154]. These results provide a wider panel of biological biomarkers for AD compared to other neurodegenerative disorders. Interestingly, many clinical trials have been started in the recent years to investigate the clinical relevance of EVs as new biomarkers for diagnosis or drug response in AD. In 2017, the University Hospital in Lille started to recruit participants to analyze levels of Tau in EVs derived from AD patient CSF (ClinicalTrials.gov Identifier: NCT03381482). In 2019, the University of Oxford began to study if the drug JNJ-40346527 can block the colony stimulating factor-1 receptor which is responsible for the regulation of microglial cells, in order to change the activity or the number of activated microglial cells in the brain. To find evidence of this change, both free protein biomarkers and the number of EVs are monitored. In the same year, the National Institute of Aging in Baltimore started a clinical trial to investigate the ability of the molecule empagliflozin, which is an antidiabetic drug, to elevate ketone levels and boost neuronal health, thus delaying the onset and progression of cognitive impairment. To this aim, Egan and colleagues isolated from plasma both total and neuronal-origin EVs to analyze if an increase in ketone bodies may upregulate IGF-1 and insulin cascades in non-diabetic individuals. These studies, only partially concluded, suggest a novel approach to identifying non-invasive diagnostic and prognostic biomarkers of neurodegeneration and provide novel insights into the clinical tools available to follow the progression of AD compared to other neurodegenerative disorders.

### PD

The urgency of identifying novel biomarkers of PD arises from the fact that the clinical symptoms as motor function alterations appear decades after the onset of neurodegeneration. At that time, a loss of a significant number of neurons has already occurred, jeopardizing any type of therapeutic intervention aimed at preserving cell viability. To date, many studies have found high levels of α-synuclein within BDEs in plasma samples of PD patients compared to healthy controls. Additionally, a direct correlation between vesicular α-synuclein levels and disease severity has been demonstrated [155]. For instance, α-synuclein levels are consistently and stably elevated in PD patients even at the early or advanced stage, allowing discrimination of PD from other syndromes (e.g., atypical parkinsonian syndrome) or healthy subjects [156]. Overall, these data support the notion that increased levels of α-synuclein, in L1CAM-containing neuronal EVs isolated from biological fluids, may be considered as a useful biomarker for early PD diagnosis, as well as a predictive marker of motor dysfunction progression in PD. Moreover, the levels of α-synuclein, phosphorylated Tyr-181 Tau, and phosphorylated insulin receptor substrate-1 in L1CAM-immunocaptured EVs isolated from plasma of PD patients, appear as useful biomarkers of cognitive prognosis [157]. Other potential biomarker candidates have been found enriched in PD-derived EVs compared to controls, such as the phosphorylated Ser-1292 Leucine-Rich Repeat Kinase 2 (LRRK2), a soluble cytoplasmic protein often found associated with intracellular membranous organelles including mitochondria, lysosomes, and endosomes [158]. The same trend has been observed for DJ-1, a highly conserved dimeric protein mainly expressed in tissues with high-energy demand, like testis, Langerhans’ islets, and brain. In neuronal cells, DJ-1 is involved in the regulation of transcription and neuronal protection from oxidative stress. To date, higher levels of DJ-1 have been found in PD patient-derived EVs compared to healthy subjects [159]. EVs may also transport other pivotal regulators of PD-related pathways, such as miRNAs. miRNA analysis allows the identification of miRNAs correlated with α-synuclein levels in the CSF. For instance, early-stage PD patients show low levels of miR-22-3p, as well as high levels of both miR-10b-5p and miR-151a-3p [160]. Moreover, higher levels of both miR-24 and miR-195 and decreased amount of miR-19b were found in EVs derived from serum of PD patients compared to healthy controls [161]. Furthermore, a recent study detected higher levels of miR-30c-2-3p in exosomes derived from plasma of PD patients. On the contrary, levels of miR-15b-5p, miR-138-5p, miR-338-3p, miR106b-3p and miR-431-5p are lower in PD subjects than in controls. Interestingly, gene ontology and Kyoto Encyclopedia of Genes and Genomes pathway analyses revealed that all dysregulated miRNAs targeted genes involved in dopaminergic synaptic formation, neurogenesis, and neuron protection guidance, further supporting their critical role in PD progression [162]. To date, despite meeting most of the criteria for an ideal biomarker, there is still a lack of standardized protocols regarding the use of circulating or exosomal miRNAs in clinical practice [163, 164]. In addition, the fraction of brain-derived miRNAs within the pool of circulating non-coding RNAs inside the blood is very small. This may lead to the loss of significant differences when analyzing their levels in biological fluids derived from patients affected by neurological disorders. To this regard, standardization of BDE isolation protocols and analysis of BDE-derived molecules may provide useful biomarkers for neurodegenerative diseases. At present, 4 clinical studies are currently ongoing to investigate the role of EVs as a source of PD-related biomarkers. Two of them (NCT03775447, Fox BioNet Project ExtraCellular Vesicles ECV-003 and NCT04603326, Fox BioNet Project ECV-004) are sponsored by the Michael J. Fox Foundation for Parkinson’s Research and aim at optimally isolating EVs from human CSF to detect LRRK2 levels and activity. The other two clinical trials are sponsored by Fondazione Don Carlo Gnocchi Onlus (NCT05452655 and NCT05320250). The first one tests the ability of a new set of serum biomarkers in NDEs to evaluate rehabilitative outcomes in a cohort of PD patients. The other one aims at validating, by Raman spectroscopy analysis, molecules isolated from either saliva or salivary EVs as new biomarkers for differential diagnosis between PD and atypical Parkinsonism.

### HD

In patients affected by HD, BDEs can contain mHTT, its fragments, or other proteins reflecting the conditions of donor CNS cells, highlighting exosome content as an important source of biomarkers for disease state and treatment status. Unfortunately, at present, further studies are needed to clarify the correlation of exosomal mHTT levels with disease severity and progression. Anyway, encouraging data highlight the potential to evaluate the levels of expanded RNA repeats and polyQ proteins in EVs derived from biofluids of HD patients as biomarkers of disease progression and response to therapy [122]. In addition, numerous miRNAs linked to HD have been found to be embodied in exosomes: (i) miR-214, miR-150, miR-146a and miR-125b, which are reported to be able to target both human and mouse HTT in a cell model of HD, (ii) miR-22 that regulates several HD-related proteins such as histone deacetylase 4 (HDAC4), REST corepressor 1 (Rcor1) and regulator of G-protein signaling 2 (Rgs2), (iii) miRNA-128a that interacts with HTT and is downregulated in HD mouse and HD patient brain tissues. Moreover, more than 60% of miRNAs dysregulated in the cortex of HD monkeys are of exosomal origin [165–168]. Finally, miR-124, which is highly expressed in the CNS and markedly down-regulated in brains of both HD patients and experimental models, has been found in serum exosomes, and its exosomal level has been reported as a promising biomarker for brain damage in ischemic stroke [169–171].

## Role of stem cell-derived EVs as novel potential therapeutic tools in neurodegenerative disorders

**Fig. 2:**
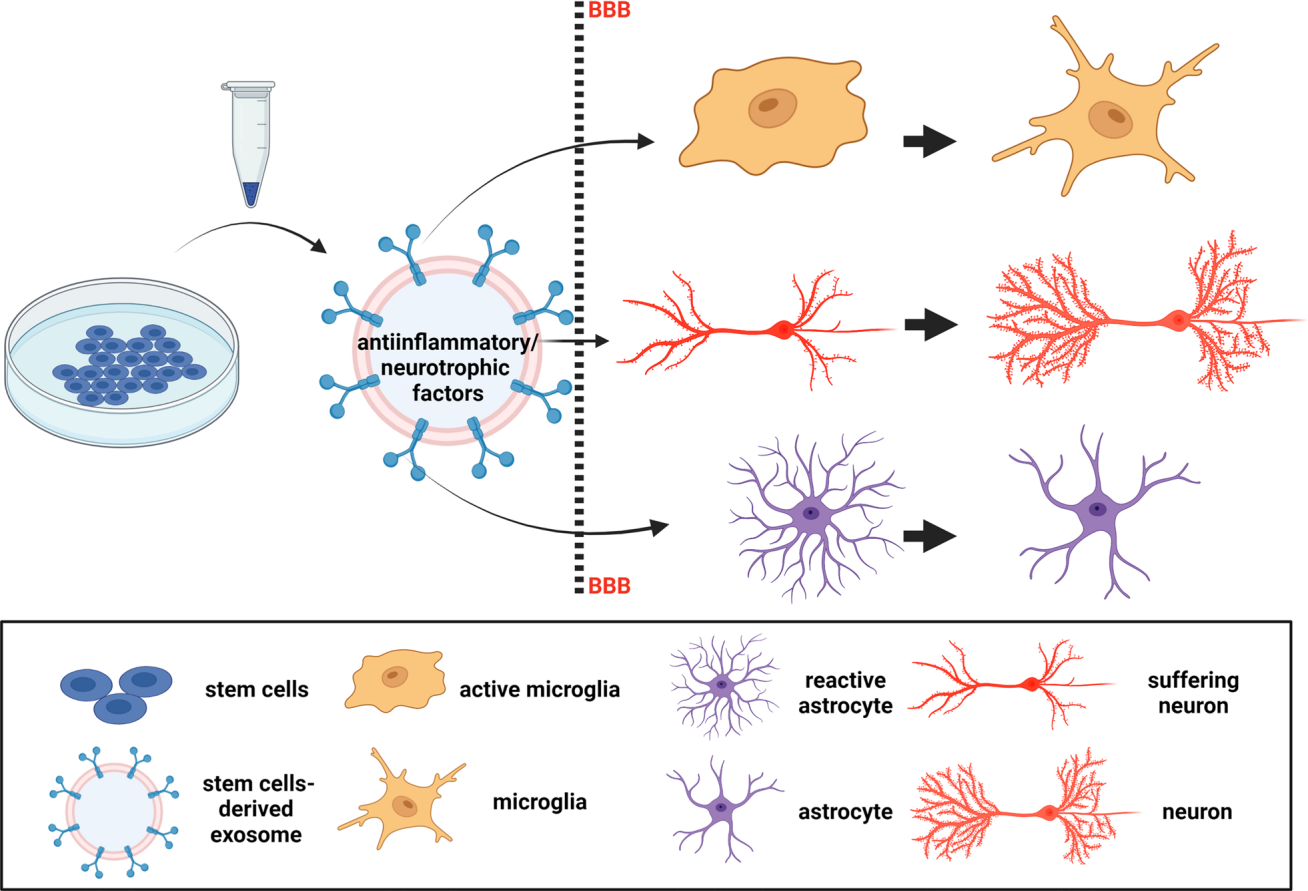
Effects of the administration of stem cell-derived EVs in neurodegenerative disorders**.** BBB, Blood Brain Barrier

Along with their potential as ubiquitous biomarkers, exosomes may enable graft-free delivery of therapeutic molecules and offer several advantages over cell-therapy. For instance, exosomes have low immunogenicity [172] and can be internalized by a variety of brain cells. Importantly, EVs have the intrinsic ability to breach biological barriers including the complex BBB, whose restrictive nature represents a significant therapeutic challenge [173]. Applications of EVs in therapies for neurodegenerative diseases embrace two main aspects: these vesicles may represent a biocompatible cargo for drug delivery and, more importantly, the stem cell-derived exosomes can offer a novel therapeutic tool against neurodegenerative disorders as a vehicle of neurotrophic and anti-inflammatory molecules potentially fostering brain plasticity [174, 175] (Fig. 2).

Like EVs from any other cells, stem cell-derived exosomes carry a cargo of miRNAs, mRNAs, proteins, and lipids. In addition, EVs containing chemokines and trophic factors originating from stem cells show immunomodulatory and reparative effects that make them promising candidates as therapeutic tools against neuroinflammation and synaptic dysfunction occurring in neurodegenerative disorders [24]. Among all kinds of pluripotent stem cells, NSC-derived exosomes have received much attention. NSC-derived EVs contain miRNAs and proteins relevant for neural regeneration, neuroprotection and neural plasticity [176]. Therefore, exogenous application of NSC-derived EVs in the adult brain in both normal and disease conditions can positively influence the intercellular microenvironment and modulate signaling pathways, potentially improving brain functions. In this regard, there is a significant interest to use NSC-derived exosomes to treat a variety of neurological disorders. There are several potential advantages of using NSC-derived exosomes compared to NSC transplantation. First, many EVs can reach different regions of the brain through intranasal administration [174, 175, 177]. Second, unlike cell therapy, the risk of developing a tumor or malignant transformation after EV administration is significantly lower since they are not nucleated cells, cannot replicate, and quickly disintegrate after releasing their cargo [178]. With the advent of new techniques, it is also feasible to generate therapeutic vesicles using large-scale cellular factories. Moreover, since EVs are stable for extended periods of time at -80 °C and for several weeks at 4 °C, they can be stored and transported easily. Thus, EVs may represent a novel frontier of cell-free therapies also for hard-to-reach tissues such as the brain. Considering the huge variety of exosome-producing cells, as well as vesicles’ cargoes, further studies are needed to fully comprehend their physiological role and to unveil their full potential in treatment and prevention of diseases. To this aim, in 2020, a clinical trial aiming to evaluate the safety and efficacy of exosomes derived from allogenic adipose mesenchymal stem cells (MSC-exos) in AD patients has been started in Shanghai, China (ClinicalTrials.gov Identifier: NCT04388982). This phase I/II clinical trial, with estimated completion date in August 2022, explores the effects of intranasal administration of different dosages of MSC-exos in the treatment of mild-to-moderate AD-related dementia in a time frame of 48 weeks. Of note, apart from neurodegenerative diseases, stem cell-derived EVs have been demonstrated to have antioxidant, anti-inflammatory, and anti-apoptotic effects in neurological, endocrine, organ-specific, and genitourinary disorders [179]. Several clinical trials have been already conducted to study the effect of EVs in the treatment of different pathological conditions such as skin wounds (Clinicaltrials.gov NCT02565264), type 1 diabetes (Clinicaltrials.gov NCT02138331), and epidermolysis bullosa (Clinicaltrials.gov NCT04173650). Anyway, at present, questions regarding the purity of different subtypes of EVs, individual responses to chronic administration, number of EVs and other details of therapeutic protocol have not been completely resolved. In addition, since EVs may be altered by the microenvironment, vesicles obtained from patients with various diseases (e.g., metabolic diseases) can potentially transfer molecules interfering with the health of host patients. Finally, before clinical application of SC-derived EVs, it would be essential to standardize protocols for their purification to avoid undesirable genetic and/or protein component transfer and deleterious immune system activation.

### AD

In the recent era, numerous studies have highlighted the potential of both NSC- and mesenchymal stem cell (MSC)-derived EV treatment to protect against synaptic loss and improve cognition in AD brains. Exosomes secreted by stem cells contain proteolytic enzymes, such as neprilysin, that effectively cleave extracellular and intracellular Aβ deposits in the brain. Accordingly, injection of bone marrow MSC-EVs into the brains of mouse models of AD decreased Aβ levels, plaque load and number of dystrophic neurites by promoting microglia-mediated phagocytosis of Aβ plaque and increasing hippocampal expression of brain-derived neurotrophic factor [148, 180].

To investigate their immune regulatory properties, EVs released by cytokine-stimulated MSCs were delivered to primary cultures of microglia and intranasally administered in a 3×Tg-AD model. In vitro, these preconditioned EVs reduced secretion of interleukins IL‐6 and IL‐1β, which play important roles in neuroinflammation and are upregulated in AD brains, and enhanced secretion of IL‐10, an anti‐inflammatory cytokine that induces the M2 polarization, associated with the formation of neuronal synapses. When administered in vivo, MSC-derived EVs were linked to a strong reduction of Iba‐1‐positive cells in the hippocampus, and enthorinal and prefrontal cortex [181]. Accordingly, recent studies have addressed the antioxidant and neuroprotective effects of human mesenchymal stem cell-derived EV (hMSC-EVs) on cultured hippocampal neurons treated with Aβ oligomers. Collectively, the results of this study demonstrated that: (1) the total number of EVs taken up by the hippocampal cells increases significantly in the presence of Aβ, (2) hMSC-EVs increase the resistance of hippocampal neurons to damage caused by Aβ, and (3) the neuroprotective effect of hMSC-EVs is correlated to the expression of enzymatically active catalase in their cargo. The authors also reported the ability of these EVs to inhibit Aβ-induced neuronal damage by modulating the astrocyte-related inflammatory responses and decreasing ROS production [182].

In addition to MSC, different stem cell-derived EVs have been shown to counteract the impairment of cognitive functions in AD experimental models through various mechanisms including the reduction of both intracellular and extracellular Aβ deposition. In recent years, numerous studies have highlighted the potential of NSC-derived exosomes in ameliorating neurodegeneration at both molecular and behavioral levels. Apodaca et al. demonstrated that systemic administration of human NSC-derived exosomes restored fear extinction memory consolidation and reduced anxiety-related behaviors in the 5×FAD AD mouse model. EV treatment restored homeostatic levels of circulating pro-inflammatory cytokines, protected against synaptic loss, improved cognition, and significantly reduced dense core amyloid-beta plaque accumulation and persistent microglial activation, which is detrimental to neuronal survival and cognitive function [183]. Li et al. investigated the effect of NSC-derived exosomes in APP/PS1 mice on cognitive behavior, mitochondrial function, sirtuin1 (SIRT1) expression, synaptic function and morphology, Aβ level, and inflammatory response. Although Aβ levels were not altered, the NSC-derived EVs rescued cognitive deficits in APP/PS1 mice, enhanced mitochondrial function, SIRT1 activation and synaptic activity, decreased inflammatory response, and rescued cognitive deficits in mouse models of AD [184], suggesting the capacity of exosomes to influence AD pathological environment through both Aβ-dependent and -independent mechanisms. Finally, intracerebral injection of exosomes secreted from hippocampal NSCs protects against the synaptotoxic action of Aβ οligomers, decreasing their binding to synapses in hippocampal slices and preventing LTP and memory deficits [185].

### PD

In the last years, the efficiency of various novel approaches, including stem cell therapy and gene therapy, have been evaluated for PD treatment [186]. Many studies have investigated and highlighted the potential therapeutic properties of the secretome derived from stem cells, mainly focusing on stem cell-derived EVs isolated from conditioned media and used in both in vitro and in vivo experimental models of PD. For instance, MSC-derived exosomes were discovered to rescue dopaminergic neurons in 6-OHDA mouse model of PD [187]. Stem cell-derived exosomes have been also demonstrated to promote neural differentiation through regulation of endogenous miRNAs and the transfer of exogenous miRNAs, and to carry beneficial miRNAs that are able to reduce neuroinflammation in PD animal models. MiR-21 and miR-143 in MSC-derived exosomes are found to play a significant role in immune modulation and neuronal death. Moreover, the delivery of MSC-derived exosomes containing miR-133b, one of the miRNAs downregulated in PD, can promote neurite outgrowth both in vitro and in vivo experimental models of PD [188]. Finally, exosomal miR-17-92 cluster promotes neurogenesis and oligodendrogenesis and improves neuronal function in the ischemic boundary zone of rats subjected to transient middle cerebral artery occlusion [189]. In addition to MSC, NSC-derived exosomes are now recognized as a fundamental source of cell therapy for PD. EVs isolated from human NSCs have been demonstrated to exert a protective effect on PD pathology both in a 6-hydroxydopamine (6-OHDA)-induced in vitro model and in an in vivo PD mouse model, where they reduce intracellular reactive oxygen species, and counteract the activation of apoptotic pathways*.* Moreover, NSC-derived exosomes carry anti-inflammatory factors and specific miRNAs (i.e., hsa-mir-182-5p, hsa-mir-183-5p, hsa-mir-9, and hsa-let-7) involved in cell differentiation, which contribute to decreased neuronal loss [190]. Therefore, understanding how the miRNAs from stem cell-derived exosomes interact with the cells and molecules in PD is of great importance.

### HD

Nowadays, alternative therapies are intensively pursued to find an effective treatment to prevent and ameliorate HD symptoms, including those based on stem cells or stem cell-derived exosomes. To date, very few studies have investigated the therapeutic effects of stem cell-derived EVs in HD. Lee et al. demonstrated that administration of EVs derived from adipose-derived stem cells (hASCs) decreased intracellular mHTT aggregates in an in vitro model of HD [191]. The authors reported that hASCs secreted exosomes containing neurotrophic factors able to counteract abnormal apoptotic protein levels and to reduce mitochondrial dysfunction by increasing the levels of both PGC-1 and phosphorylated CREB proteins. Accordingly, neurotrophic factor levels have been inversely related to HD progression and approaches stimulating the expression of BDNF appear to delay the onset of cognitive decline in experimental models of HD [192, 193]. However, further studies are necessary to clearly establish the effectiveness of stem cell-derived EVs in experimental models of HD.

## Conclusions

The knowledge of the pathophysiology of neurodegenerative disorders still has many grey areas, such as the availability of diagnostic and therapeutic tools which are often scarce and insufficient. The lack of reliable biomarkers and personalized treatments is a severe limitation for early and accurate patient management in neurodegenerative diseases triggering cognitive decline and dementia. Furthermore, drug delivery and treatments are limited by the BBB. Exosomes, a subtype of endogenous nanoscale vesicles, play a key role in cell signaling through the transmission of genetic information and proteins to nearby and distant cells. Because of their ability to cross the BBB, EVs may represent potential biomarkers of the CNS disorders and they can be used as a therapeutic carrier to deliver molecules into the brain. At the same time, numerous studies have highlighted the involvement of EVs in the onset and progression of several disorders in the brain. Exosomes secreted by glial cells or neurons in an unhealthy microenvironment affect the interactions and thus the physiology of brain cells by transmitting miRNAs, proteins, and lipids, and carrying oxidative and inflammatory signals from cell to cell. Furthermore, vesicles may also deliver pathogenic materials (e.g., protein aggregates) from one cell to another, worsening the progression of diseases like AD, PD and HD. In addition, stem cell-derived exosomes (e.g., exosomes derived from neural stem cells and mesenchymal stem cells) have demonstrated great capacity to provide therapeutic benefits and a fundamental question is whether exosomes originating from different types of stem cells possess different protective/therapeutic effects in pathological environments. Of note, once the key signaling components of SC-derived EVs (e.g., surface receptors/proteins) are identified, they can be engineered to facilitate drug delivery in the brain and even specific targeting of different types of neural cells. In conclusion, exosomes are emerging as crucial messengers in the brain, and further studies are needed to fully clarify their roles in both physiological and pathological conditions.

## Data Availability

Not applicable.

## Abbreviations

AD: Alzheimer’s disease
BBB: Blood–brain barrier
BDE: Brain-derived exosome
CNS: Central nervous system
CSF: Cerebral spinal fluid
EV: Extracellular vesicles
HD: Huntington’s disease
mHTT: Mutant Huntingtin
MSC: Mesenchymal stem cell
NSC: Neural stem cell
PD: Parkinson’s disease
